# Application Value of High Resolution Magnetic Resonance Imaging in Preoperative Evaluation of Non-melanoma Skin Cancer

**DOI:** 10.2174/0115734056360576250731135230

**Published:** 2025-08-26

**Authors:** Xiaoqiong Li, Xinghua Ji, Yanjun Liang, Weibin Dai, Yueyou Peng, Yanfeng Meng

**Affiliations:** 1 Department of Radiology, Taiyuan Central Hospital, No.1 Dongsan Daoxiang, 030009, Taiyuan, Shanxi Province, China; 2 Department of Orthopedics, Shanxi Academy of Medical Sciences, 99 Longcheng Street, 030032, Taiyuan City, Shanxi Province, China; 3 Plastic Surgery (Wound Repair), Taiyuan Central Hospital, No.1 Dongsan Daoxiang, 030009, Taiyuan, Shanxi Province, China

**Keywords:** High-resolution magnetic resonance, Basal cell carcinoma, Cutaneous squamous cell carcinoma, Preoperative evaluation, Skin tumor, OCT

## Abstract

**Introduction:**

Conventional skin tumor examination shows inherent limitations in accurately assessing tumor depth. HR-MRI offers superior soft tissue resolution and a comprehensive evaluation of skin cancer.

**Methods:**

Patients confirmed by pathological diagnosis as non-melanoma skin cancer from January 2021 to December 2023 were enrolled. Patients in Group 1 received both HR-MRI and tumorectomy, while those in Group 2 received tumorectomy only. The exclusion criteria include patients with contraindications to magnetic resonance examination. MRI sequences included T1WI, T2WI, and T2WI fat suppression, and a dynamic contrast-enhanced(DCE) scan. The advantages of different sequences in evaluating the level of invasion were independently assessed by two radiologists. The advantages of different sequences in evaluating the level of invasion were independently assessed by two radiologists. Tumor size, shape, invasion, and dynamic curves were measured in a corresponding sequence. And tumor signal intensity was recorded in different sequences. For each group, the number of postoperative tissue sections, sections with positive margins, and cases of secondary surgery were recorded. For Group 1, pathological invasion levels were also recorded.

**Results:**

89 cases of non-melanoma skin cancer were collected, including 69 basal cell carcinoma (BBC) and 20 squamous cell carcinoma (SCC). There were 25 patients in group 1 and 59 patients in group 2. T1WI showed mainly isointensity or hypointensity for BCC and SCC. T2WI showed predominantly hyperintense, and T2WI with fat suppression all showed hyperintense. T2WI effectively showed the relationship between tumors and nearby structures, while fat-suppressed T2WI highlighted tumor contours. The positive rate of pathological sections and the rate of secondary resection in group 1 and group 2 were 9.7% and 20%, 23.1% and 44.1%. There was a higher consistency between tumor invasion levels observed by MRI and pathological results in the first group (p>0.75)

**Discussion:**

Advancements in skin tumor diagnosis and treatment reveal that some tumors penetrate deeper than traditional methods can detect, prompting interest in MRI research. HR-MRI, known for its excellent soft tissue resolution, proves useful in outlining tumors and determining their location, particularly with the T2 fat-suppressed sequence. The T2WI sequence effectively assesses skin invasion, aligning well with pathological findings, and this significantly reduces the need for subsequent surgical interventions.. This underscores HR-MRI's value as a preoperative tool. However, the study's small sample size is a limitation, and future research will include more cases for deeper insights.

**Conclusion:**

Skin HR-MRI is valuable for non-melanoma skin cancer, providing accurate preoperative tumor scope assessment, and reducing the rate of secondary surgeries.

## 1 INTRODUCTION

Skin cancer is broadly classified into melanoma and nonmelanoma types, both of which represent prevalent malignant tumors, particularly among the elderly population. Among nonmelanoma skin cancers (NMSCs), basal cell carcinoma (BCC) and squamous cell carcinoma (SCC) are the most common subtypes. The incidence of cutaneous malignancies in Europe and North America has demonstrated a consistent upward trend, which aligns with global demographic aging patterns [1-8] and consequently positions skin cancer as being the most frequently diagnosed malignant neoplasm in Western countries [9]. According to 2019 cancer spectrum statistics for the Chinese population, NMSC has become the fifth most common malignant tumor occurring among elderly individuals [10]. The etiology of NMSC is closely linked to factors such as ultraviolet radiation exposure, ionizing radiation, and chronic inflammation [11, 12], which predispose individuals to a greater likelihood of developing these cancers in sun-exposed areas, particularly in the facial and neck regions [13]. Surgical intervention remains the cornerstone of treatment, with Mohs micrographic surgery (MMS) being the most widely utilized surgical technique [14-17]. Compared with alternative treatment modalities, MMS has significantly improved two-year survival rates in elderly patients with NMSC [14]. Consequently, preoperative visual assessments to determine the extent and depth of tumor infiltration are critical for optimizing surgical planning and prognostic evaluation [18].

Common diagnostic techniques for skin tumors include dermoscopy [19-22], reflectance confocal microscopy (RCM) [23-30], optical coherence tomography (OCT) [31, 32], and high-frequency ultrasound [32, 33]. Although magnetic resonance imaging (MRI) is not traditionally the first choice for examining skin tumors, its exceptional soft tissue contrast makes it a valuable tool in clinical practice. Research on high-resolution magnetic resonance imaging (HR-MRI) of the skin has been conducted as early as 1987 [34]; however, progress has decreased due to limitations in spatial resolution. With continuous advancements in MRI equipment and technology, imaging quality has significantly improved, thereby reigniting interest in the application of skin MRI [35-42]. Conventional methods for skin tumor examinations demonstrate certain limitations, such as restricted depth penetration (which can result in inspection blind areas) and the potential for the occurrence of tumor deformations during high-frequency ultrasound due to compression. In contrast, high-resolution MRI offers distinct advantages over traditional techniques. It provides superior depth and breadth in a single examination, thus enabling a more comprehensive assessment of the tumor and its relationship with surrounding structures. Additionally, MRI is less influenced by examiner-related variables, thereby ensuring a more standardized and objective procedure. Moreover, MRI supports multiparametric scanning, which offers valuable insights into various biological characteristics of the lesion, such as the presence of hemorrhage or fat. For skin tumors, the significance of imaging evaluation is based on its ability to preoperatively assess the size, extent, and depth of the tumor, which plays a crucial role in surgical planning. Due to their limitations regarding penetration depth and coverage, traditional imaging modalities may introduce certain inaccuracies in preoperative planning. Magnetic resonance imaging (MRI) serves as an excellent supplement to address these issues regarding traditional imaging.

In this study, the contribution of HR-MRI to Mohs surgery for nonmelanoma skin cancer was evaluated.

## 2 MATERIALS AND METHODS

### 2.1 Subjects

This study was a prospective cohort study. Patients with skin tumors who presented to Taiyuan Central Hospital (the Dermatology Department and Radiology Department) between January 2021 and December 2023 were enrolled. The inclusion criterion involved cases of nonmelanoma skin cancer that were confirmed *via* biopsy. Exclusion criteria included: (1) patients with contraindications to magnetic resonance imaging (MRI); and (2) patients with contraindications to surgical procedures. High-resolution magnetic resonance imaging of the skin was conducted with the informed consent of the patients. An additional inclusion criterion involved patients with pathologically confirmed NMSC who underwent surgical treatment. According to whether HR-MRI was performed, surgical patients were randomly divided into two groups: Group 1 consisted of patients who received skin HR-MRI examinations prior to surgery, whereas Group 2 comprised patients who did not undergo skin HR-MRI examinations before their surgical procedures. The study received approval from the hospital's ethics committee (2020007), and informed consent was obtained from all of the participating patients.

The pathological evaluations of the cases were interpreted at the Dermatopathology Laboratory of Taiyuan Central Hospital, and the radiological evaluations were performed at the Radiology Department of Taiyuan Central Hospital. Image analysis was independently performed by two experienced radiologists.

### 2.2 Magnetic Resonance Imaging

The scanning protocol utilized a Siemens Skyra 3.0T magnetic resonance scanner, whereby an appropriate coil was utilized for the anatomical region of interest. The scanning sequences included TSE T1 with a TR of 628 ms and a TE of 10 ms; TSE T2 with a TR of 3000 ms and a TE of 87 ms; and TSE FST2 with a TR of 3500 ms and a TE of 87 ms. The field of view (FOV) was set to 120×120 mm, with a slice thickness ranging from 2 to 4 mm, a slice spacing of 10%, and pixel dimensions of 0.2×0.2×2.0 cm. A T1 VIBE dynamic contrast-enhanced (DCE) scan was conducted utilizing a gadolinium-based contrast agent, which was administered *via* a high-pressure injector at a flow rate of 2 ml/s into the elbow vein. The dosage of the contrast agent was 0.1 mmol/kg. Subsequently, 30 ml of saline solution was injected, thereby completing the enhancement process. Following the injection of the contrast agent, five consecutive dynamic enhanced images were acquired, with each phase exhibiting a scanning duration of 76 seconds.

### 2.3 Image Analysis

The size, margin, signal intensity, enhancement curve characteristics, and involved layers (including the epidermis, dermis, subcutaneous fat, fascia, muscle, and periosteum layers) of the tumors were assessed based on magnetic resonance imaging data. Tumor size was quantified using the T2-weighted imaging (T2WI). In Group 1, the tumor size was delineated according to HR-MRI images (the A1 line).

Two radiologists independently measured the tumor sizes (including transverse diameter, longitudinal diameter, and depth), analyzed signal characteristics across all of the imaging sequences (such as T1-weighted imaging, or T1WI; T2-weighted imaging, or T2WI; and T2-weighted imaging with fat suppression, or FS-T2WI), and identified the deepest layer of tumor invasion.

### 2.4 Surgical Methods

The tumor size was assessed *via* visual inspection, and the mean diameter was subsequently calculated. In Group 2, the tumor extent was determined using standard preoperative evaluations and delineated with the A2 line. The surgeon subsequently marked the B1 and B2 lines along the periphery of the A1 and A2 lines, respectively, at distances varying from 0.3 cm to 1.5 cm, based on the tumor size and its pathological classification.

#### 2.4.1 Tissue from the Tumor Center was Excised for Pathological Examination

Parallel tissue slices were excised along the demarcated A1 and A2 lines, with the tumor tissue extending to the subcutaneous fat layer.

Parallel tissue slices were excised along the B1 and B2 lines. The resected tumor tissue was subsequently divided into several smaller fragments, each with a diameter of ≤ 1 cm.

Following paraffin embedding and hematoxylin and eosin (HE) staining, the tumor cells within the tissue were microscopically examined to assess if the tumor had been completely removed. If residual tumor cells were detected, additional parallel tissue slices were excised until the tumor was completely removed.

### 2.5 Statistical Analysis of Pathological Sections

The number of tissue sections, the number of sections with residual tissues at the margins (positive), and the number of cases requiring secondary resections for each group were recorded. Furthermore, the degree of pathological invasion in patients who underwent magnetic resonance imaging examinations was documented.

### 2.6 Study Size

The sample size for this study was calculated based on the primary outcome of the negative margin rate. Assuming a negative margin rate of 90% in the HR-MRI group and a rate of 75% in the non-HR-MRI group, along with a significance level (α) of 0.05 and a statistical power (1-β) of 0.80, a minimum of 50 participants per group was needed to detect a 15% difference *via* the chi-square test. To account for potential dropouts or incomplete data, we aimed to enroll 60 participants per group. However, due to the limited number of eligible patients that were available during the study period, the final sample size included 25 participants in the HR-MRI group and 59 participants in the non-HR-MRI group. Post hoc power analysis confirmed that the study had adequate power (≥0.80) to detect significant differences in the negative margin rates and secondary resection rates between the two groups.

### 2.7 Statistical Methods

The repeatability of the magnetic resonance image interpretations by the two radiologists was evaluated using the chi-square test, with statistical significance defined as p > 0.05. Quantitative data, including age, average tumor diameter, and distances of the A and B lines, were compared between Group 1 and Group 2 using the independent samples *t* test, with statistical significance set at *p* < 0.05. The Mann-Whitney U test was used to analyze the count data and to specifically compare the percentages of negative tissue sections between Group 1 and Group 2, with statistical significance indicated by *p* < 0.05. A chi-square test was applied to analyze the ordinal data and was used to compare differences in secondary resection rates between Group 1 and Group 2, as well as discrepancies between the levels of tumor invasion identified *via* magnetic resonance imaging and the pathological findings. Statistical significance was established at *p* < 0.05. All of the statistical analyses were performed using SPSS software version 26.0.

## 3 RESULTS

A total of 89 cases of malignant skin tumors were collected, consisting of 69 BCC patients and 20 SCC patients. Among the BCC patients, 30 were male, and 39 were female, with an average age of 64.8 years. Among these cases, 64 occurred in the maxillofacial region, whereas 5 were located on the trunk. For SCC, there were 8 male and 12 female patients, with an average age of 71.8 years being observed. Sixteen of these cases occurred in the maxillofacial region, whereas 4 were located on the limbs (Table **1**). Skin HR-MRI scans were performed on a total of 30 patients, comprising 16 males and 14 females (aged between 38 and 89 years, with an average age of 63.7 years). Among the lesions, 28 were located in the maxillofacial region, whereas 2 were observed in the limbs, all of which were identified as SCCs. Specifically, the BCC group comprised 21 patients, including 11 males and 10 females (with ages ranging from 38 to 88 years and an average age of 64 years); additionally, the SCC group consisted of 9 patients, including 5 males and 4 females (with ages ranging from 57 to 89 years and an average age of 71 years).

There was no statistically significant difference in the tumor signal characteristics (Table **2**) and measurements assessed (Table **3**) by the two radiologists. The measured transverse diameter, longitudinal diameter, and depth values of the BCCs on MRI were 14.53±8.88 mm, 13.00±10.64 mm, and 4.59±2.83 mm, respectively. For the SCCs, the corresponding measurements were 21.89±19.07 mm, 19.11±14.61 mm, and 7.44±4.48 mm, respectively. Two patients diagnosed with each tumor type did not undergo TSE FST2 sequence scanning. In BCC patients, the T1WI signal intensity was isointense in 13 patients, moderately hyperintense in 5 patients, and hypointense in 3 patients. On T2WI, the signal intensity manifested as hyperintensity in 9 patients, moderate hyperintensity in 6 patients, asymmetric hyperintensity in 4 patients, and isointensity in 2 patients. On FS-T2WI, the signal intensity revealed hyperintensity in 13 patients, asymmetric hyperintensity in 6 patients, and moderate hyperintensity in 1 patient. For SCC patients, the T1WI signal appeared to be isointense in 6 patients, moderately hyperintense in 1 patient, and hypointense in 2 patients. On T2WI, the signal intensity was presented as moderate hyperintensity in 5 patients, asymmetric hyperintensity in 1 patient, and isointensity in 3 patients. On FS-T2WI, the signal intensity exhibited hyperintensity in 5 patients, moderate hyperintensity in 1 patient, and moderate hyperintensity in 2 patients (Table **4**). The signal characteristics of SCC and BCC are consistent with the findings of the study by Masaya *et al*. [39] A total of 16 patients with BBC and 6 patients with SCC were included in the DCE scans. Among these patients, the enhancement curve exhibited a “plateau pattern” in 9 BBC patients and a “washout pattern” in 7 BBC patients. For SCC, 1 patient exhibited an “inflow pattern”, and 5 patients exhibited a “plateau pattern”.

The morphology of BCC on MRI manifested as a superficial-like appearance in 8 patients, a nodular-like appearance in 4 patients, a hill-like appearance in 4 patients, a cauliflower-like appearance in 1 patient, and a volcano-like appearance in 4 patients. In contrast, SCC MRI revealed a superficial-like appearance in 1 patient, a nodular-like appearance in 4 patients, a creeping growth-like appearance in 2 patients, and an irregular-like appearance in 2 patients.

The preferred sequences for displaying both tumors included FS-T2WI in 20 instances and T2WI in 1 instance where FS-T2WI was not performed. The sequences providing more detailed information for assessing the level of invasion were the T1WI in 6 instances and the T2WI in 24 instances (Table **5**). The interobserver agreement between the two observers regarding signal characteristics and the level of tumor invasion was assessed using the chi-square test. The Kappa value exceeded 0.7, thereby indicating relatively high consistency. For tumor size measurements, a paired t-test was performed, and the resulting P value was less than 0.05, thereby further confirming a high level of agreement between the observers.

A total of 84 patients underwent tumorectomy. Group 1 comprised 25 patients, including 13 males and 12 females (aged 38 to 89 years, with an average age of 64.52 years). Within this group, there were 19 cases of BCCs and 6 cases of SCCs. The mean diameter of the tumors (as measured *via* visual inspection) was 1.54 cm, and the average distance between lines A and B was 0.75 cm. A total of 145 tissue slices were obtained after surgery, which included 14 positive margins (where the pathological assessment of the tissue section identified residual tumor cells) and 131 negative margins (where the pathological assessment of the tissue section identified no residual tumor cells), thus resulting in a positivity rate (the ratio of positive margins to the total number of slices) of approximately 9.7% and a negativity rate (the ratio of negative margins to the total number of slices) of approximately 90.3%. Additionally, 5 patients underwent secondary resection (with complete tumor removal not being achievable in one operation), thereby resulting in a secondary resection rate of 20%.

In Group 2, there were 59 patients, including 22 males and 37 females (aged 32 to 87 years, with an average age of 66.63 years). Among these patients, 48 cases of BCC and 11 cases of SCC were diagnosed. The mean diameter of the tumors (as measured *via* visual inspection) was 1.32 cm, and the mean distance between lines A and B was 0.68 cm. A total of 302 tissue sections were obtained after surgery, among which 70 positive margins and 232 negative margins were obtained, thus resulting in a positivity rate of approximately 23.1% and a negativity rate of approximately 76.9%, respectively. There were also 26 patients who required secondary resections, which resulted in a secondary resection rate of 44.1%. No statistically significant differences were observed between Group 1 and Group 2 in terms of sex, age, visually assessed tumor size, distance between lines A and B, or pathological results (p > 0.05) (t test). However, significant differences were observed in both the negative margin rate and the secondary resection rate (p < 0.05) (Mann-Whitney U test) (Table **6**). Specifically, Group 1 demonstrated a lower positivity rate at surgical margins and a lower secondary resection rate compared with Group 2. The level of tumor invasion (as assessed *via* MRI) aligned with the pathological findings in 22 patients, whereas discrepancies were observed in 3 patients. The agreement between the MRI and pathological results was excellent, with a Kappa value exceeding 0.75 (p > 0.75) (chi square test) (Table **7**). It was also found that there was a high consistency between the magnetic resonance results and the pathological results in the evaluation of tumor thickness in the study by Reiko *et al*. [37].

## 4 DISCUSSION

NMSC is a prevalent malignant tumor, with the incidence of BCC being approximately twice that of SCC. These cancers predominantly occur in sun-exposed areas, with the maxillofacial region being the most commonly affected area, followed by the trunk, lower limbs, upper limbs, neck, and scalp [43]. The skin, which is the largest organ of the human body, possesses an average surface area of approximately 2.0 m2. Despite its extensive coverage, skin thickness varies significantly and ranges from 0.5 mm to 4 mm [44]. The thicknesses of the different skin layers also considerably vary, with significant differences being observed among the layers based on orders of magnitude of 100 μm for the epidermis and stratum corneum, millimeters for the dermis, and centimeters for the hypodermis [45, 46]. Consequently, imaging methods that are capable of distinguishing among the skin layers require a resolution of at least 100 μm. Common imaging techniques for evaluating skin tumors include dermoscopy, RCM, and OCT [47-49], as well as high-frequency ultrasound [50]. However, these methods are constrained by their limited penetration depths, thereby reducing their efficacy in assessing tumors with deep invasion. For lesions with severely compromised surfaces, ultrasound examination may exhibit certain challenges, and compression during the procedure can alter the tumor's shape, thus potentially affecting the accuracy of the morphological assessment. Additionally, the accuracy of these imaging results is highly dependent on the operator's expertise.

As the understanding of the diagnosis and treatment of skin tumors has advanced, the observed invasion depths of certain skin tumors have surpassed the detection capabilities of traditional examination methods. Consequently, research on skin MRI has been increasingly pursued, with several advantages being documented, including the notion that HR-MRI demonstrates superior soft tissue resolution. Numerous studies have confirmed that high-resolution MRI meets the necessary resolution criteria for the examination of skin tumors [35, 36, 45, 51-56]. This imaging modality can distinctly delineate the epidermis, dermis, subcutaneous fat, fascia, blood vessels, hair follicles, and other anatomical structures. For example, on T2WI, the epidermis appears as a linear, slightly hyperintense structure, the dermis is visualized as an isointense tissue layer, and the deeper subcutaneous adipose tissue exhibits a hyperintense signal. Furthermore, the image clearly delineates hypointense fascial and vascular structures within the tissue. Specific MRI sequences are capable of differentiating between the papillary dermis and the reticular dermis [53]. Ye *et al*. utilized HR-MRI to evaluate the efficacy of hair loss treatment. In addition to clearly distinguishing among the layers of the scalp, HR-MRI also enabled the visualization of hair follicle count and depth, thereby providing a precise assessment of the therapeutic outcomes for hair loss [57]. Multiparameter imaging offers comprehensive biological information. This technique does not exert pressure on the lesion, thereby preventing any deformation of the tumor and allowing for a more precise assessment of its extent. Additionally, minimal requirements exist regarding the surface condition of the lesion, which ensures that the examination results remain unaffected by surface disruptions or infections. This method also accommodates a greater depth and breadth of the examination, thus indicating that it is particularly advantageous for evaluating lesions with extensive scopes and deep infiltrations. Structures such as deeper muscle tissues and bones can also be clearly visualized. In addition to allowing for the precise delineation of the relationship between the tumor and each anatomical layer, this method allows for the evaluation of deeper pathological conditions. Compared with other diagnostic modalities, high-resolution MRI not only provides superior resolution but also ensures comprehensive coverage of the examination area. Michael *et al*. conducted an MRI study on the perineural spread of squamous cell carcinoma in the maxillofacial region, and they reported a sensitivity of 89% and a positive predictive value (PPV) of 97% in detecting facial nerve invasion [58].

Current studies on skin MRI have predominantly utilized small-diameter single-loop coils to achieve ultrahigh-resolution imaging [34, 39, 40, 47, 51-59]. However, surface coils demonstrate three significant disadvantages [60]. First, when considering patient discomfort and cooperation issues, the surface coils must be directly fixed over the lesion area, which can cause discomfort due to local compression. This issue is particularly pronounced in the maxillofacial region, where the scanning procedure and resulting compression may lead to involuntary facial movements. Additionally, elderly patients often exhibit lower levels of cooperation, thus resulting in a higher rate of scan failure. Second, the inspection scope of the surface coils, as well as the heterogeneity in the scanning signal, is considerably limited, with a significant disparity being observed in signal intensity between regions proximal to and distal from the coil center. This limitation hinders the evaluation of lesions exhibiting extensive scopes and deep infiltrations. Third, there is inadequate coverage for large lesions related to surface coils. In cases where the lesion area exceeds the coverage provided by the surface coil, the comprehensive visualization of the tumor is unachievable, which significantly constrains the preoperative assessment of the extent of the tumor. In contrast, the use of a standard coil for scanning the relevant areas (although potentially leading to a loss of image resolution) remains adequate for diagnostic purposes. In our study, the smallest measured tumor diameter was 2 mm. Depending on the pathological types and sizes of the tumors, the surgical resection margin can be radially extended by 2–10 mm beyond the tumor edge [17, 61-64]. Moreover, the pixel dimensions of the images that were obtained using the standard coil were 0.2 × 0.2 × 2.0 cm. Despite some loss of resolution, this scale was adequate for meeting the planning requirements for surgical resection. Consequently, in most cases, the use of a standard coil was sufficient. However, surface coils can be employed for more detailed observations of fine structures to obtain additional information.

**Fig. (1):**
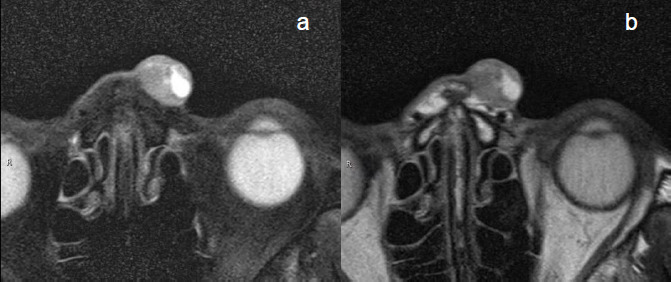
MRI of BCC in left nasal root showed hyperintense on T2WI fat suppression with clear profile (**a**); the tumor infiltrated into subcutaneous fat on T2WI (**b**). Cystic structures can be observed within the lesion.

Different imaging sequences with varying signal intensities exhibit distinct diagnostic efficacies for various diseases. Previous studies have significantly underestimated the diagnostic value of FS-T2WI in the assessment of skin tumors [37, 62]. This study demonstrated that all of the cases exhibited hyperintensity on FS-T2WI, whereas the subcutaneous fat appeared to be hypointense. Compared with adjacent tissues, the tumors demonstrated marked signal contrast, thereby facilitating a more precise delineation of the contours of the tumors. This finding is consistent with the conclusions of Xu *et al*. regarding the evaluation of skin tumors across different imaging sequences [65]. FS-T2WI demonstrated distinct advantages in identifying small or superficially growing tumors with linear patterns and could complement the use of other sequences in defining tumor extent. However, due to challenges in differentiating structures such as the subcutaneous fascia and blood vessels, FS-T2WI is not suitable for assessing invasion depth. On FS-T2WI, tumors typically exhibit pronounced hyperintensity or isointensity, whereas subcutaneous fat appears to be hypointense. The significant signal contrast between the tumors and surrounding tissues enhances boundary visualization, particularly for tumor localization (especially when the tumor is located). Nevertheless, FS-T2WI experiences issues in differentiating subcutaneous structures such as the fascia and blood vessels, thereby limiting its efficacy in evaluating invasion depth (Fig. **1a**). On T2WI, tumors are depicted with relatively hyperintense signals against a background of adipose tissue, whereas structures such as the fascia, vascular tissue, dermis, and epidermis appear to be hypointense. This approach enables more straightforward observations of the relationships between tumors and various layers of the skin and subcutaneous structures (Fig. **1b**). Previous studies by Kang *et al*. [62] and Reiko *et al*. [37] have demonstrated high concordance between the tumor invasion levels identified *via* MRI and pathological findings. Consistent with these findings, we also observed a high concordance (Kappa > 0.75) between the tumor invasion levels identified on T2WI images and the postoperative pathological results. However, in 6 patients in this study, the imaging findings did not align with the pathological results. These discrepancies primarily involved tumors located at the interface between two skin layers, where the accurate assessment of deeper layer involvement is challenging. In such cases, imaging evaluations may overestimate the extent of invasion. Additionally, melanin exhibits characteristic hyperintense signals on T1WI, thereby aiding in the differentiation of melanoma-originating tumors.

**Fig. (2):**
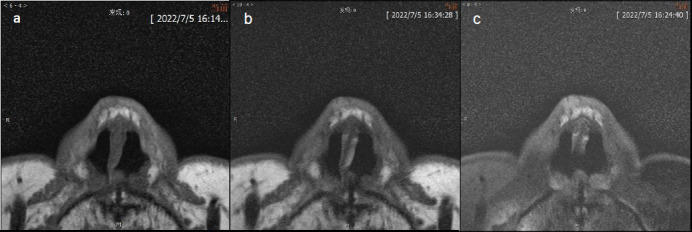

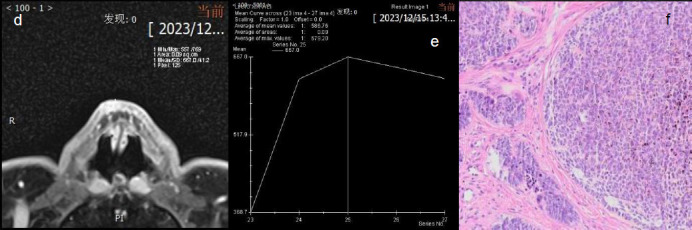
MRI of BCC in nasal tip. The tumor appeared isointense on T1WI (**a**), hyperintense on T2WI (**b**) and FST2WI (**c**). The dynamic enhancement images showed significant enhancement (**d**) with outflow pattern in dynamic enhancement curve (**e**). The pathology confirmed the tumor was BCC (10×) (**f**).

**Fig. (3):**
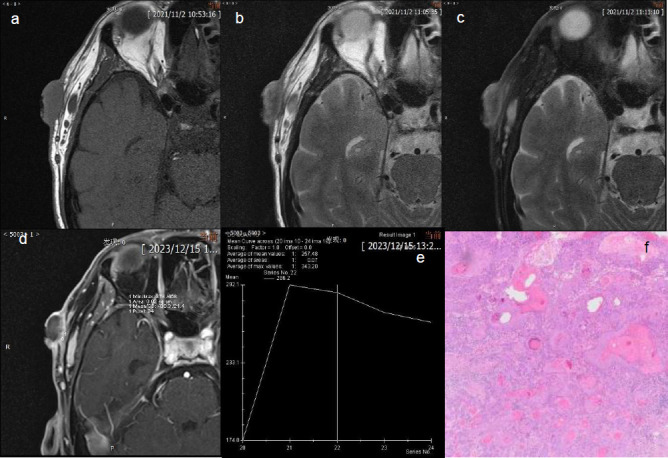
MRI of SCC in right temporal area. The tumor appears isointense on T1WI (**a**), asymmetric hyperintensity on T2WI (**b**) and asymmetric hyperintensity on FST2WI (**c**). The dynamic enhancement imaging showed ring enhancenment pattern with ulcer (**d**). The dynamic enhancement curve showed an outflow pattern (**e**). The pathology confirmed SCC (10×) (**f**).

**Fig. (4):**
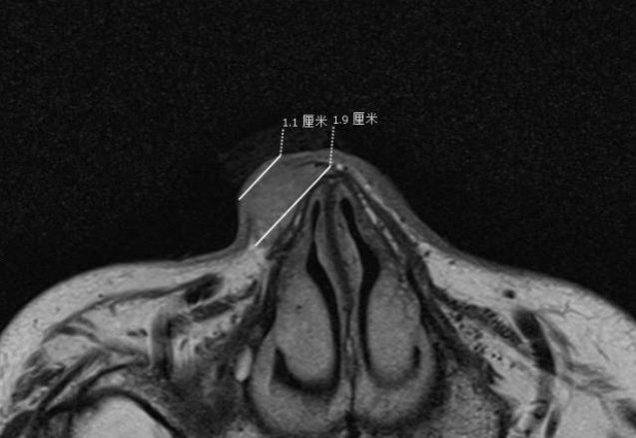
Shows BCC on the nose, with surface diameter of approximately 1.1cm and deep diameter of approximately 1.9cm, indicating that the tumor depth extends beyond the surface.

BCC Figs. (**2a**-**f**) and SCC Fig. (**3a**-**f**) exhibit predominantly isointense signals on T1WI (Figs. **2a**,**3a**), whereas on T2WI (Figs. **2b**,**3b**) and FS-T2WI (Figs. **2c**,**3c**), they predominantly display hyperintense signals. The signal characteristics of these two tumor types are similar, thus leading to challenges regarding their differentiation. In this study, 6 cases of BCC exhibited heterogeneous intensity on T2WI within the tumor (Figs. **2d**,**3d**). This phenomenon may be attributed to the inflammatory mucoid characteristics of the BCC stroma, which comprises a complex mixture of components, including mucus, lymphocytes, fibroblasts, collagen, and other elements. Notably, two cases of nodular-type BCC exhibited distinct cystic structures with hyperintense signals on T2WI and well-defined boundaries (Fig. **1**). This observation may be explained by the tendency of nodular BCC to form large mucous lakes or cystic cavities, which is a characteristic feature of this subtype [66]. The imaging morphology of BCC demonstrates variations, with typical presentations of smooth outer margins and minimally localized invasive features on MRI. Some patients exhibit linear lesions within the epidermis, whereas others exhibit nodular lesions. Notably, there was minimal disparity observed between the transverse extent of deeper tissue involvement and that of the tumor itself. In some instances, the lesions exhibited significant depth, which was characterized by an “iceberg” growth pattern in which external manifestations were minimal compared with extensive internal involvement (Fig. **4**). Preoperative MRI evaluation is crucial for assessing such morphological features. BCC originates in the basal layer of the epidermis, which is relatively superficial and exhibits a relatively low degree of malignancy. In this study, the mean depth of the BCC samples was 4.6 mm, with the maximum depth not exceeding 10 mm. Typically, BCC is confined to the dermis and extends only to the superficial layer of the subcutaneous fat. However, periocular lesions (where subcutaneous fat is absent) are more likely to involve the orbicularis oculi muscle. The MRI morphology of some SCC cases can resemble that of BCC, with some nodules exhibiting smooth outer margins, which complicates differentiation. However, SCC exhibits more invasive behavior, which is characterized by deeper infiltration and racemose-like growth patterns. In this study, the average depth of the SCC was approximately 7.45 mm, with the maximum depth reaching 15 mm and extending into the adjacent bones. Dynamic contrast-enhanced MRI revealed that BCC demonstrated a plateau-type enhancement curve in 9 patients and a washout-type curve in 7 patients (Figs. **2e**, **f**). In contrast, SCC exhibited an inflow-type curve in 1 patient and a plateau-type curve in 5 patients (Figs. **3e**, **f**). These enhancement patterns are generally consistent with those observed in other malignant tumors.

NMSC predominantly occurs in the maxillofacial region, whereby it significantly impacts the aesthetic appearance and potentially invades critical structures such as the nerves and muscles, thereby compromising facial function [67]. Surgical interventions aim not only to achieve complete tumor excision but also to preserve functional anatomical structures. Mohs micrographic surgery (MMS) is the preferred surgical technique for this type of cancer [68, 69] and involves the initial removal of the visible tumor, followed by meticulous excision of the underlying tissue in the thin layers. The excised tissue layers are sectioned into small portions (each smaller than 1 cm^2^ in size) and mapped by using normal tissues as references for microscopic examinations. Sequential thin-layer removal is performed until no tumor tissue remains at the surgical margins. This technique integrates histopathological examinations, thereby ensuring complete tumor excision while minimizing the risk of recurrence. Additionally, this technique maintains consistent surgical margins and conserves as much normal tissue as possible, which creates optimal conditions for subsequent skin grafting. This approach promotes improved wound healing and achieves aesthetically favorable outcomes [67-71]. Slow Mohs micrographic surgery (sMMS) employs formalin fixation and paraffin embedding of the tissues. Although this method enhances the accuracy of pathological sectioning, it is time-consuming and may delay surgical site healing [71]. In cases where the tumor cannot be entirely excised in a single procedure, subsequent surgeries may further compromise wound healing and reduce patient tolerance, particularly in elderly individuals. Accurate preoperative delineation of the extent of the tumor and infiltration depth can improve the success rate of primary resection and reduce the need for secondary surgery. With its high resolution and extensive coverage, HR-MRI provides excellent preoperative guidance for surgical planning. Furthermore, it serves as an important supplement to skin imaging, whereby it combines the advantages of other diagnostic modalities with unique and irreplaceable benefits.

**Fig. (5):**
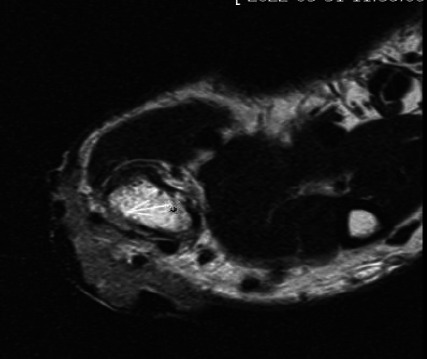
Squamous cell carcinoma on the dorsum of the thumb, deeply infiltrating the thumb periosteum.

Based on the imaging analysis, the morphological characteristics of the two types of tumors were demonstrated to be significantly different. In certain instances, the extents of the lesions visible to the naked eye were considerably smaller than that of the subcutaneous lesions (Fig. **4**). For example, SCC lesions tended to more deeply invade the tissues and potentially involve the periosteum (Fig. **5**). Conventional dermatological examinations may not accurately assess the depth of the lesion invasion, which can potentially lead to an underestimation of the extent of the lesion and an increase in the likelihood of requiring multiple surgical interventions. HR-MRI offers extensive coverage, which enables comprehensive visualization of the entire tumor and precise delineation of its boundaries. This finding is also consistent with the results of the study by Tang *et al*. regarding the preoperative application value of MRI in skin tumors [40]. This scenario significantly aids in surgical planning, as supported by the results of previous studies.

## 5 LIMITATIONS

However, this study had several limitations, including a small sample size and the inability to comprehensively represent all of the imaging characteristics of NMSC, which may restrict the generalizability of the findings. Additionally, the lack of comparative analysis with other diagnostic modalities further limits the scope of this research. In subsequent studies, we plan to collect more cases and conduct comparative evaluations with other imaging techniques to further validate and expand on our understanding of the imaging features of NMSC.

## CONCLUSION

In conclusion, HR-MRI of the skin offers several advantages, including high resolution (which allows for the visualization of skin details), a wide examination range (which enables the assessment of deep tumor invasion), and avoidance of effects of factors such as lesion surface ulceration. These features make it a promising diagnostic tool for skin tumors, thereby serving as a complementary examination method. Moreover, it provides a more accurate evaluation of tumor extent and facilitates the strategic planning of surgical margins, thereby optimizing surgical outcomes.

## Figures and Tables

**Table 1:** Basic information of the case.

-	-	**BCC**	**SCC**
Age	-	64.83±12.38	71.8±9.57
Gender	Male	30	8
	Famale	39	12
Location of occurrence	Maxillofacial	64	16
	Trunk	5	0
	Limbs	0	4

**Table 2:** Consistency of tumor characteristics by two radiologists.

	**Kappa-value**	***P*-*value***
T1WI (BCC)	0.901	0.001
T2WI (BCC)	0.773	0.001
FST2 (BCC)	0.811	0.001
T1WI (SCC)	0.727	0.023
T2WI (SCC)	0.824	0.001
FST2 (SCC)	0.742	0.004
Better display contour (BCC)	1.000	0.001
Better display contour(SCC)	1.000	0.003
Better display relationship with adjacent structures (BCC)	0.774	0.001
Better display relationship with adjacent structures(SCC)	0.727	0.023
Shape in the images(BCC)	0.813	0.001
Shape in the images(SCC)	1.000	0.001

Kappa test was used to analyze the consistency between two radiologists' observations. The P-values for all items in the table were less than 0.05, indicating a relatively high level of agreement between the two radiologist' observations.

**Table 3:** Consistency evaluation of tumor diameter measurement by two radiologist.

	**Radiologist 1**	**Radiologist 2**	**T**	**P**
BCC				
Transverse diameter	14.94±8.84	14.53±8.88	1.951	0.069
Longitudinal diameter	13.17±10.52	13.00±10.64	0.717	0.484
Depth	4.76±2.51	4.59±2.83	0.765	0.455
SCC				
Transverse diameter	22.67±17.86	21.89±19.07	0.563	0.589
Longitudinal diameter	18.56±14.31	19.11±14.61	-0.887	0.401
Depth	7.89±4.91	7.44±4.48	1.180	0.272

Paired t-test used to assess the consistency of tumor size measurements by two radiologists, both P-values were greater than 0.05, indicating that the tumor size measurements by the two radiologists are consistent.

**Table 4:** Characteristics of tumor signals.

**Sequence**	**Signal**	**BCC**	**SCC**
T1WI	Isointensity	13	6
	Moderate hyperintensity	5	1
	Hypointensity	3	2
T2WI	Hyperintensity	9	0
	Moderate hyperintensity	6	5
	Asymmetric hyperintensity	4	1
	Isointensity	2	3
FST2	Hyperintensity	13	5
	Asymmetric hyperintensity	6	1
	Moderate hyperintensity	1	2

**Table 5:** Advantages of different sequences.

**Sequence**	**Better Display Contour**			**Better Display Relationship with Adjacent Structures**		
	**T1**	**T2**	**FST2**	**T1**	**T2**	**FST2**
BCC	0	1	20	6	15	0
SCC	0	0	9	0	9	0
total	0	1	29	6	24	0

**Table 6:** Differential analysis of two groups of patients.

**Variables**	**1 (n = 25)**	**2 (n = 59)**	**χ^2^/t**	**p**
Gender, n (%)			1.017	0.313*
Male	13 (52)	22 (37)		
Female	12 (48)	37 (63)		
Age, Mean ± SD	64.52 ± 14.02	66.63 ± 11.4	0.664	0.511#
Mean diameter, Mean ± SD	1.32 ± 1.08	1.54 ± 1.2	0.826	0.411#
Spacing of lines A and B, Mean ± SD	0.98 ± 0.39	0.75 ± 0.28	0.76	0.45#
Negative rate of section,, Median (Q1,Q3)	100 (90, 100)	80 (45, 100)	-3.09	0.002&
Secondary resection, n (%)			3.907	0.048*
No	10 (40)	39 (66)		
Yes	15 (60)	20 (34)		
Pathology, n (%)			0.068	0.794*
BCC	19 (76)	48 (81)		
SCC	6 (24)	11 (19)		

*P adopts chi-square test; #p adopts t-test; &p adopts rank-sum test. There was no statistical significance in gender, age, average diameter, distance between A and B lines, and pathological differences between Group 1 and Group 2 (p>0.05). However, there were statistically significant differences in negative rate and whether a second resection was performed (P<0.05).

**Table 7:** Consistency evaluation of imaging and pathology.

	**Level of invasion**					**Total**	**χ^2^**	**p**
	**Dermis**	**The Subcutaneous Fat Layer**	**Myofacial**	**Muscle**	**periosteum**			
MRI imaging	3	12	1	8	1	25	1.268	0.867
Pathology	6	10	1	7	1	25		

There was good consistency between MRI image observation and pathological invasion level (p>0.75).

## Data Availability

The data of current study are available from corresponding author, [Y.M], on a reasonable request.

## LIST OF ABBREVIATIONS

NMSCs: Nonmelanoma skin cancers
BCC: Basal cell carcinoma
SCC: Squamous cell carcinoma
MMS: Mohs micrographic surgery
RCM: Reflectance confocal microscopy
OCT: Optical coherence tomography
HR-MRI: High-resolution magnetic resonance image

## STANDARDS OF REPORTING

STROBE guidelines were followed.
